# The *Laetiporus* polyketide synthase LpaA produces a series of antifungal polyenes

**DOI:** 10.1038/s41429-020-00362-6

**Published:** 2020-08-21

**Authors:** Paula Sophie Seibold, Claudius Lenz, Markus Gressler, Dirk Hoffmeister

**Affiliations:** Pharmaceutical Microbiology, Friedrich Schiller University, Jena, Germany

**Keywords:** Multienzyme complexes, Fungal physiology

## Abstract

The conspicuous bright golden to orange-reddish coloration of species of the basidiomycete genus *Laetiporus* is a hallmark feature of their fruiting bodies, known among mushroom hunters as the “chicken of the woods”. This report describes the identification of an eight-domain mono-modular highly reducing polyketide synthase as sole enzyme necessary for laetiporic acid biosynthesis. Heterologous pathway reconstitution in both *Aspergillus nidulans* and *Aspergillus niger* verified that LpaA functions as a multi-chain length polyene synthase, which produces a cocktail of laetiporic acids with a methyl-branched C_26_–C_32_ main chain. Laetiporic acids show a marked antifungal activity on *Aspergillus* protoplasts. Given the multiple products of a single biosynthesis enzyme, our work underscores the diversity-oriented character of basidiomycete natural product biosynthesis.

## Introduction

**Fig. 1:**
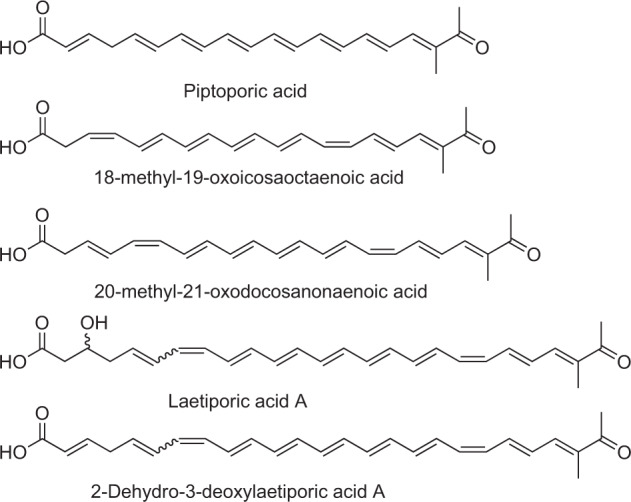
Structures of basidiomycete polyenes. For laetiporic acid A, the predominant isomer is shown (*cis*-configured double bond C_7_–C_8_)

Non-terpenoid polyenes are a remarkable class of biologically active basidiomycete natural products. These compounds with up to ten conjugated carbon–carbon double bonds have been attributed to chemical defense: piptoporic acid (Fig. 1), a polyene from *Piptoporus australiensis* with seven double bonds in conjugation deters fungivorous larvae from feeding on the fruiting bodies [1, 2]. More recently, 18-methyl-19-oxoicosaoctaenoic acid and 20-methyl-21-oxodocosanonaenoic acid (Fig. 1) of a taxonomically undescribed stereaceous basidiomycete, preliminarily referred to as BY1, were shown to inhibit pupation of larvae [3]. Biosynthetically, the respective compounds are polyketides and were instrumental in gaining first insight in polyene biogenesis in Basidiomycota as the BY1 multi-domain highly reducing polyketide synthase (HR-PKS) PPS1 was functionally reconstituted in *Aspergillus niger* as heterologous host [4].

The intense, conspicuous orange color is the signature feature of specimens of the *Laetiporus sulphureus* species complex, i.e., the “chicken of the woods” fungi. These are brown-rotting bracket mushrooms that have a European and North American distribution and which are commonly found on oak, eucalypt, or willow trees. The coloration is conferred by a blend of polyenes. In previous works, Weber et al. elucidated the structure of laetiporic acid A and its 2 dehydro-3-deoxy derivative (Fig. 1) [5, 6], i.e., two non-terpenoid polyenes that possess a C_26_ main chain and share the 1-methyl-2-oxo-propylidene moiety with the above basidiomycete polyenes yet show ten conjugated double bonds. The same authors detected even longer putative polyene products, laetiporic acids B and C (with C_28_ and C_30_ main chains, respectively) by liquid chromatography and mass spectrometry.

To learn more about the structural diversity of fungal polyenes, including the as yet largely uninvestigated biosynthesis of non-aromatic polyketides in basidiomycetes, we built upon the above previous *Laetiporus*-related results. Herein, we describe the *L. sulphureus* enzyme LpaA as a multi-chain length polyene synthase. Heterologous pathway reconstitution in two independent recombinant *lpaA*-expressing *Aspergillus* species led to a polyene profile similar to that found in *L. sulphureus* mycelium and fruiting bodies. We demonstrate that LpaA, i.e., a single HR-PKS, produces a series of compounds with C_26_–C_32_ main chain lengths.

## Materials and methods

### General experimental procedures

Semipreparative HPLC was performed on an Agilent 1260 instrument, equipped with a diode-array detector, UHPLC-MS runs were done on an Agilent 1290 Infinity II chromatograph, interfaced to an Agilent 6130 single quadrupole mass detector. HR-ESI-MS spectra were recorded in positive mode on a Thermo Scientific Exactive Orbitrap instrument. UV/Vis spectra were recorded from *λ* = 200–700 nm with diode array detectors connected with the respective chromatographs, chromatograms were extracted at *λ* = 450 nm.

### Microbial strains and growth conditions

*Escherichia coli* XL1-blue was used for routine cloning and was cultivated in LB supplemented with 50 µg ml^−1^ carbenicillin, if required. *Laetiporus sulphureus* (s.l.) JMRC SF012599 was provided by the Jena Microbial Resource Collection (JMRC) and was routinely maintained on MEP medium (per liter: malt extract 30 g, soytone peptone 3 g, agar 18 g) for 14 days at 25 °C. To produce laetiporic acids, *L. sulphureus* was cultivated on 150 YPD agar plates (per liter: yeast extract 10 g, soytone peptone 20 g, d-glucose 20 g, agar 18 g) at 20 °C for 14–21 days. Fruiting bodies of *L. sulphureus* were collected in Jena, Germany, on willow trees along the Saale river, in September 2019. *Aspergillus* strains used for transformation were *A. niger* ATNT16Δ*pyrG*x24 [7] and *A. nidulans* FGSC A4.

*A. niger* transformants tPS01 and tPS02 were cultivated on *Aspergillus* minimal medium agar plates (AMM) [8] supplemented with 5 mM l-glutamine at 30 °C for 5–7 days. Media for ATNT16Δ*pyrG*x24 were supplemented with 10 mM uridine. To produce laetiporic acids in recombinant *A. niger*, transformants tPS01 (vector control) and tPS02 (polyene producer) were pre-cultivated overnight in 30 Erlenmeyer flasks, each filled with 50 ml YPD medium, at 30 °C and 140 rpm. The main culture was a 30 × 1 l fermentation (AMM containing 200 mM d-glucose and 50 mM l-glutamine), inoculated with 50 ml pre-culture each. To induce *lpaA* expression, 30 mg l^−1^ doxycycline was added after 18 h, and cultivation was continued for additional 48 h. *A. nidulans* FGSC A4 and mutant tMG01 were maintained on AMM plates supplemented with 5 mM l-glutamine at 37 °C for 3 days. Plates for tMG01 were supplemented with 0.1 µg ml^−1^ pyrithiamine hydrobromide. To produce laetiporic acids in *A. nidulans*, the strains were cultivated in 100 l auto-inducing AMM, prepared with 200 mM ethanol and 10 mM d-glucose as carbon sources, at 30 °C and 140 rpm for 72 h. Details on fungal strains are given in Supplementary Table S1. *Aspergillus* conidia were harvested with 10 ml sterile water and the suspension was filtered by a cell strainer (40 µm, EASYstrainer). Media were inoculated at a titer of 1 × 10^6^ conidia per milliliter.

### cDNA cloning and construction of *lpaA* expression plasmids

*L. sulphureus* mycelium was grown in liquid YPD medium at 20 °C and 140 rpm for 7 days, harvested, and ground under liquid nitrogen. RNA was isolated using the SV Total RNA Isolation Kit (Promega). Residual genomic DNA was digested by Baseline-ZERO DNase (Biozym). Reverse transcription was carried out with anchored oligo-dT_18_ primers and RevertAid Reverse Transcriptase (ThermoFisher). The *lpaA* coding sequence was PCR-amplified from the first strand reaction, using the oligonucleotides oCL46 and oCL47 (Supplementary Table S2), using method A (Supplementary Table S3). The gel-purified fragment was ligated into pJET1.2 (Thermo) to yield plasmid pCL10 (Supplementary Table S4) which was sequenced (GenBank accession number MT304701) to verify accurate amplification and then served as template for subsequent PCRs. A tag-free version (8190 bp) was expressed in *A. nidulans*, while in *A. niger*, a gene for a hexahistidine fusion protein was used (8229 bp).

The *lpaA* coding sequence was PCR-amplified (method B, Supplementary Table S3) from pCL10 using oMG459 and oMG460 (Supplementary Table S2) in order to introduce *Pac*I sites at either end of the fragment. The *A. niger* expression vector pSMX2-URA [7], allowing for doxycycline-inducible gene expression, was modified by PCR-mediated ligation (oligonucleotides oMG457/oMG458) to incorporate a *Pac*I restriction site in the multiple cloning site to create vector pPS01 (Supplementary Tables S2, S4). Both the insert and pPS01 were restricted with *Pac*I and ligated to create the *lpaA* expression vector pPS03. Plasmids pPS01 (vector) and pPS03 (*lpaA* expression plasmid) were used to transform *A. niger*.

To construct an alcohol-inducible *lpaA* expression vector, the vector backbone of plasmid pMD03 [9] as well as the *lpaA* coding sequence inserted in plasmid pCL10 were amplified (method B, Supplementary Table S3) using oligonucleotides oMG468/oMG469 and oMG471/oMG472, respectively. Both fragments were ligated using the NEBuilder HiFi DNA Assembly Cloning Kit (NEB) to yield *lpaA* expression plasmid pMG49, which was used to transform *A. nidulans*. Details of plasmids are described in Supplementary Table S4.

### Transformation of *Aspergillus* species

Protoplast transformation of *A. niger* and *A. nidulans* was carried out as previously described [7, 9]. In brief, protoplasts were obtained by incubation of mycelium with VinoflowPro (1.1 g per 20 ml volume) for 4 h in YAT buffer (0.6 M KCl, 50 mM maleic acid, pH 5.5) and 10 µg of plasmid DNA (pPS01 and pPS03 for *A. niger*, pMG49 for *A. nidulans*) were used for polyethylene glycol-mediated transformation. *A. niger* transformants (tPS01 and tPS02) were selected by uracil prototrophy, while *A. nidulans* transformants (tMG01) were selected by pyrithiamine resistance in presence of 0.1 µg ml^−1^ pyrithiamine. Integration of the *lpaA* gene was confirmed by PCR (Supplementary Table S3, methods C and D).

### UHPLC analysis of laetiporic acids

Methanolic crude extracts of mycelia from Aspergilli and *Laetiporus* mycelia and carpophores were centrifuged, filtered, and were subjected to UHPLC measurements. Method B (Supplementary Table S5) was used for initial screening of extracts of positive transformants or selection of fractions containing laetiporic acids during the purification procedure (see below). Method B was also applied for polyene quantification in growth inhibition assays.

### Purification of laetiporic acids

One-hundred and fifty *Laetiporus* agar plates were diced, lyophilized, and extracted with acetone (3 × 5 l). *Aspergillus* mycelia were collected, washed with water, and lyophilized. Mycelia were ground to a fine powder and extracted six times with methanol and subsequently twice with acetone (50 ml per 1 g dry biomass and extraction).

*Aspergillus* and *Laetiporus* extracts were filtered through cellulose round filters and evaporated to dryness. The dry residue was dissolved in 2 l water and repeatedly extracted with a total of 12 l ethyl acetate. The organic phase was evaporated. The residue was dissolved in 400 ml methanol, and 40 ml aliquots were subjected to size exclusion chromatography on Sephadex LH-20 (60 × 4 cm) with methanol as eluent. Three fractions (FI-III) were obtained, containing laetiporic acid (LA)-A, LA-B in F-I, LA-C in F-II, and LA-D and traces of other derivatives in F-III. All fractions were subjected to reversed phase semi-preparative HPLC, using methods C (F-I and F-II) and D (F-III) (Supplementary Table S5). Isolated compounds were lyophilized and dissolved in methanol. Final work up was accomplished under slightly basic conditions (method E for LA-A and LA-B, method F for LA-D, Supplementary Table S5).

To test for photoisomerization, 100 µg ml^−1^ of *Aspergillus*-produced laetiporic acids A_1_, B_1_, or B_2_, respectively, or laetiporic acid A_1_ from *L. sulphureus* were continuously exposed to light for 24 h (or in the dark for control). After exposure, the solutions were chromatographically analyzed by UHPLC-MS (method A, Supplementary Table S5).

### Growth inhibition assays

Cultivations were carried out in triplicate at 30 °C and 140 rpm with a conidial titer of 1 × 10^6^ per ml in 30 ml medium. *A. niger* tPS01 and tPS02 were cultivated in AMM (+200 mM d-glucose and 50 mM l-glutamine) for 42 h. Doxycycline hydrochloride (0, 7.5, 15, 30, 60, or 120 µg ml^−1^) was added immediately after inoculation. *A. nidulans* strains were cultivated in AMM without glucose, but with 200 mM ethanol as sole carbon source (inducing condition) for 72 h. For delayed gene expression, d-glucose (0, 2.5, 5, 10, or 50 mM) was added prior to inoculation. AMM with 200 mM d-glucose (repressing condition) served as negative control. After cultivation, the mycelium was lyophilized, weighted, ground to a fine powder, and extracted with 1 ml methanol for 5 min in an ultrasonic bath. After centrifugation (10 min, 13,000 *g*), an aliquot of 5 µl was subjected to HPLC analysis (method A, Supplementary Table S5) and polyene signals were manually integrated (*λ* = 450 nm, *t*_R_ = 4–8 min). Polyenes were quantified against a calibration curve with respective SEC-purified polyenes as authentic reference standards.

### Protoplast toxicity assays

To obtain polyene-containing and control extracts, *A. nidulans* was cultivated in AMM (+10 mM d-glucose and 200 mM ethanol) at 30 °C and 140 rpm for 72 h. Lyophilized mycelium (5 g) was ground to a powder and extracted five times with 200 ml methanol. Extracts were purified via size exclusion chromatography with Sephadex LH-20 as described above. Fractions were analyzed with UHPLC-MS, and fractions containing laetiporic acids were pooled for tMG01 and added in concentrations from 31 µg ml^−1^ to 4 mg ml^−1^. As negative control, appropriate SEC fractions of *A. nidulans* wild type were pooled accordingly. The eluates were dried under reduced pressure and residues were suspended in sterile YAT buffer.

To produce protoplasts, *A. nidulans* mycelium was filtered, washed with sterile YAT buffer, and incubated in lysis solution (1.3 g VinoTaste Pro (Novozymes), 0.1 g lysing enzymes from *Trichoderma harzianum* (Sigma), 0.1 g Yatalase (Takara), in 20 ml YAT buffer) at 30 °C and 70 rpm for 3 h. Protoplasts were filtered through sterile Miracloth, washed with YAT buffer, counted, and diluted to a final titer of 2 × 10^4^ protoplasts per ml. 100 µl (2 × 10^3^ cells) of the cell suspension were gently mixed with the same volume of extracts (*A. nidulans* FGSCA4 or tMG01), and protoplasts were incubated on ice for 3 h. Suspensions were carefully plated on osmotic AMM plates (with 1.2 M sorbitol, 100 mM d-glucose, 20 mM l-glutamine, pH 6.5) and incubated at room temperature for 96 h. Colony forming units from four independent experiments were counted.

## Results

### Identification of HR-PKS genes in *L. sulphureus*

**Fig. 2:**
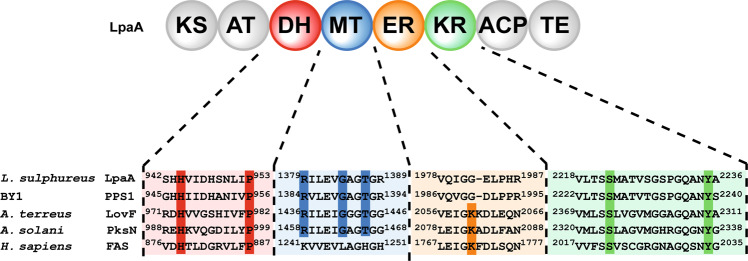
Domain setup of LpaA. Domain abbreviations: β-keto synthase (KS), acyltransferase (AT), dehydratase (DH), methyltransferase (MT), enoyl reductase (ER), β-keto reductase (KR), acyl carrier protein (ACP), thioesterase (TE). Below: amino acid sequence alignments of the active site of the respective domains. Conserved residues are highlighted with vertical color-coded bars. BY1 mushroom polyene synthase PPS1 (NCBI accession #: KX819293.1); *A. terreus* LovF: *Aspergillus terreus* lovastatin diketide synthase (Q9Y7D5.1); *A. solani* PksN: *Alternaria solani* alternapyrone synthase (BAD83684.1); *H. sapiens* FAS: human fatty acid synthase (NP_004095.4). A MT domain is not present in the human FAS. The ER domain is inactive in LpaA and PPS1. A TE domain is not present in LovF and PksN

The chemical structures of the laetiporic acids from *L. sulphureus* and the polyenes isolated from the BY1 mushroom differ in their chain length, yet share a 1-methyl-2-oxo-1-propylidene moiety and a shifted conjugated double bond system, i.e., the double bonds are positioned within the formal acetate units. We therefore used the sequence of PPS1, the only known basidiomycete polyene synthase, as query to browse the published genome of *L. sulphureus* [10]. We identified two near-identical HR-PKS genes, now collectively referred to as *lpaA*, that both encode proteins of 2729 amino acids (95% identical and 97% similar amino acids) with a corresponding molecular mass of 296 kDa. The predicted domain architecture ketosynthase–acyltransferase–dehydratase–methyltransferase–enoylreductase–ketoreductase–acyl carrier protein–thioesterase (KS–AT–DH–MT–ER^0^–KR–ACP–TE, Fig. 2) is consistent with the enzymatic requirements to biosynthesize a methyl branched polyene. The protein LpaA shared 68% identity (81% similarity) to PPS1. Analyses of amino acid sequences identified conserved canonical active sites in all domains, except the ER domain. Consistent with the not fully reduced laetiporic acids, we assumed that the ER domain in LpaA was most likely not functional. Usually, a lysine residue serves as a proton donor during reduction of the enoyl [11]. This role was evident in mammalian fatty acid synthases (K^1771^) [12], or fully reducing PKSs, such as LovF from *Aspergillus terreus* (K^2060^) [13] and *Alternaria solani* PKSN (K^2082^) [14]. However, in LpaA, and PPS1 alike, this lysine residue is replaced by glycine (G^1982^ and G^1990^), respectively, indicating a structural rather than a catalytic function of the ER (Fig. 2).

### Construction of recombinant, *lpaA*-expressing *Aspergillus niger*

**Fig. 3:**
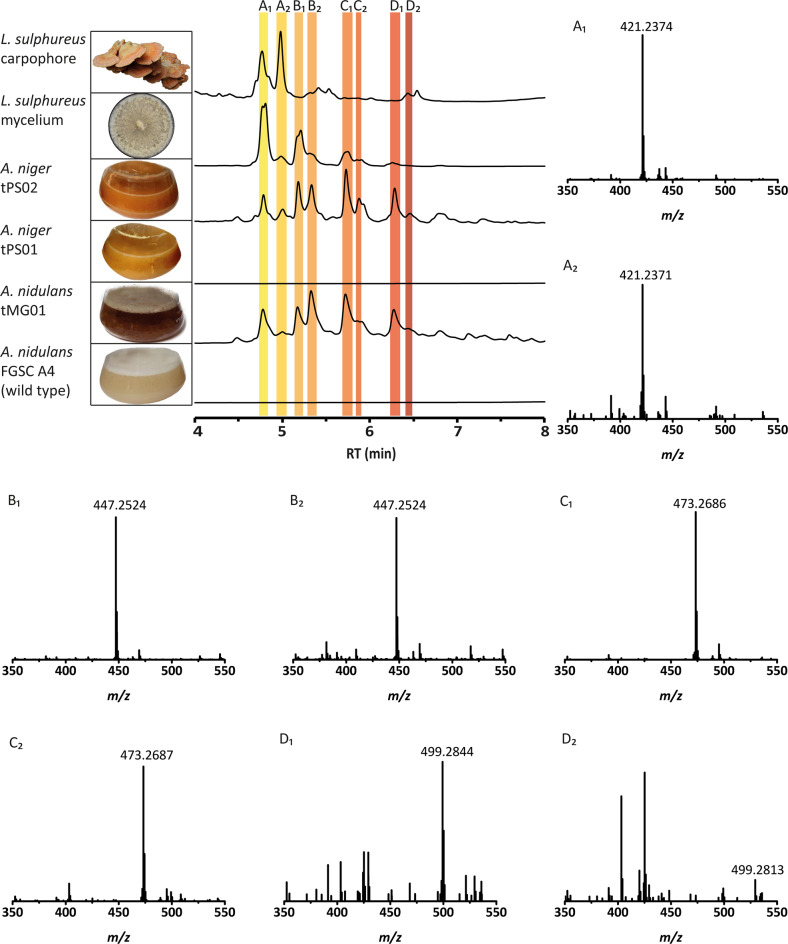
UHPLC profiles of metabolic extracts from cultures of *L. sulphureus*, *lpaA*-expressing *Aspergillus* strains and their respective control strains. Representative pictures of the fruiting bodies or cultures are presented (left). UHPLC profiles (top to bottom) of methanolic extracts from *L. sulphureus* carpophores, vegetative mycelium of *L. sulphureus*, *A. niger* tPS02 (*lpaA*-expressing), *A. niger* tPS01 (vector control), *A. nidulans* tMG01 (*lpaA*-expressing) and *A. nidulans* FGSC4 wildtype strain. Peaks for laetiporic acids A_1_–D_2_ are indicated by colored bars. Chromatograms were extracted at *λ* = 450 nm. Minor UV-absorbing compounds surrounding laetiporic acids A_1_–D_2_ are present that have not been identified, but were absent in the controls. Respective high-resolution mass spectra are given for laetiporic acids A_1_–D_2_ purified from *A. niger*. Polyene peak intensities of mass spectra range between 6 × 10^5^ (LA-D_2_) and 1.8  × 10^7^ (LA-B_1_). Please refer to Table 1 for MS² data. LA-D_2_ was produced in insufficient quantities for further analysis

*L. sulphureus* produces laetiporic acid A in fruiting bodies as well as in mycelia, as described [5] and confirmed in our study by UHPLC-MS and HR-MS (Fig. 3). To investigate the function of *lpaA*, the full-length gene was amplified from cDNA of mycelium, sequenced, and ligated to plasmid pSMX2-URA to allow for a tunable, doxycycline-dependent expression in the host *A. niger* ATNT16Δ*pyrG*x24 [7]. The fungus was transformed with a *lpaA*-expressing plasmid (pPS03) as well as an empty vector (pPS01) as control, yielding *A. niger* tPS02 (*lpaA*-expressing) and control strain *A. niger* tPS01 (Supplementary Table S1) The full-length integration of the constructs into the genome was verified by PCR (Supplementary Fig. S1). Both tPS01 and tPS02, were initially cultivated in presence and absence of doxycycline, but immediate induction led to poor growth of tPS02, while it did not impact growth of tPS01. To produce sufficient biomass for further chromatographic analysis, the cultures were induced with doxycycline (30 mg l^−1^) 18 h post inoculation. The cultivation was continued for another 48 h during which mycelium of tPS02 turned orange (Fig. 3), while both the culture supernatant and the control strain tPS01 did not change their color.

### Identification of laetiporic acids from *lpaA*-expressing *Aspergillus* strains

Mycelia of the induced transformants tPS01 and tPS02 were extracted with methanol. A subsequent UHPLC-MS analysis revealed various novel signals in tPS02 at *λ* = 450 nm, compared to the control tPS01. UV/Vis spectra of the new peaks showed absorption maxima between *λ* = 430–480 nm (Supplementary Fig. S2) and were reminiscent of the spectra of BY1 polyenes 18-methyl-19-oxoicosaoctaenoic acid and 20-methyl-21-oxodocosanonaenoic acid [4]. We therefore hypothesized the signals may reflect the presence of polyenes due to activity of LpaA in vivo. Curiously, a series of masses was found which followed a regular pattern in increments of *m/z* + 26, i.e., C_2_H_2_ (Table 1). The eight most abundant peaks (Fig. 3) were purified by size exclusion and semipreparative liquid chromatography and were finally subjected to HR-MS/MS detecting over a range between *m/z* 200 and 1400. Three detected signals are compatible with the masses of laetiporic acids A_1_ (*m/z* 421 [M + H]^+^), B_1_ (*m/z* 447 [M + H]^+^) and C_1_ (*m/z* 473 [M + H]^+^) as previously described [5]. Furthermore, we found a compound with *m/z* 499 [M + H]^+^ which we preliminarily refer to as laetiporic acid D_1_. The masses point to main chain lengths of C_26_–C_32_. Surprisingly, this set of masses appeared duplicated (laetiporic acids A_2_, (*m/z* 421 [M + H]^+^), B_2_ (*m/z* 447 [M + H]^+^), C_2_ (*m/z* 473 [M + H]^+^) and D_2_ (*m/z* 499 [M + H]^+^) which may reflect a cis/trans-isomerization of the polyene backbone. A 7-cis isomer corresponding to the 7-trans laetiporic acid A has previously been isolated from *L. sulphureus* [5, 6]. Additional confirmation that laetiporic acids had been produced in the transgenic Aspergilli came from comparative LC-MS^2^ analysis of laetiporic acid A_1_ with a standard isolated from mycelium of the original producer *L*. *sulphureus* (Figures S3 and S4) which yielded virtually identical signal patterns. Laetiporic acids were described as stable compounds [5]. To confirm these prior results, we exposed laetiporic acids A_1_, (isolated both from *Aspergillus niger* tPS02 and *L. sulphureus*), B_1_, and B_2_, respectively, to light for 24 h, or kept them in the dark for control. Chromatographic analysis did not indicate new signals, which supports the notion of laetiporic acids as stable compounds (Supplementary Fig. S5). In return, this finding suggests that the polyene diversity is an inherent feature of *Laetiporus* polyketide biosynthesis.

**Table 1:** MS and MS^2^ data of laetiporic acid pairs A_1_/A_2_–D_1_/D_2_

Compound	*t*_R_ (min)	Formula	Neutral mass [M]	Found parental ion [M + H]^+^	MS^2^ specific ions		
					Ion mass	Formula	Origin
LA-A_1_/A_2_	4.7/4.9	C_27_H_32_O_4_	420.2301	421.2374/421.2371	403.227	C_27_H_31_O_3_	[M-H_2_O + H]^+^
					385.216	C_27_H_29_O_2_	[M-2 H_2_O + H]^+^
					359.236	C_26_H_31_O	[M-H_2_O-CO_2_ + H]^+^
					343.190	C_21_H_27_O_4_	[M-C_6_H_6_ + H]^+^
					245.132	C_19_H_17_	[M-C_8_H_8_-H_8_O_4_ + H]^+^
					219.117	C_17_H_15_	[M-C_10_H_10_-H_8_O_4_ + H]^+^
					193.101	C_15_H_13_	[M-C_12_H_12_-H_8_O_4_ + H]^+^
					131.086	C_10_H_11_	[C_10_H_10_ + H]^+^
					109.065	C_7_H_10_O	[C_7_H_9_O + H]^+^
					105.070	C_8_H_9_	[C_8_H_8_ + H]^+^
					79.054	C_6_H_7_	[C_6_H_6_ + H]^+^
LA-B_1_/B_2_	5.1/5.3	C_29_H_34_O_4_	446.2457	447.2524/447.2524	429.242	C_29_H_33_O_3_	[M-H_2_O + H]^+^
					411.231	C_29_H_31_O_2_	[M-2 H_2_O + H]^+^
					369.204	C_23_H_29_O_4_	[M-C_6_H_6_ + H]^+^
					351.195	C_23_H_27_O_3_	[M-C_6_H_6_-H_2_O + H]^+^
					312.172	C_20_H_24_O_3_	[M-C_9_H_9_-H_2_O + H]^+^
					271.148	C_21_H_19_	[M-C_8_H_8_-H_8_O_4_ + H]^+^
					245.132	C_19_H_17_	[M-C_10_H_10_-H_8_O_4_ + H]^+^
					219.117	C_17_H_15_	[M-C_12_H_12_-H_8_O_4_ + H]^+^
					193.101	C_15_H_13_	[M-C_14_H_14_-H_8_O_4_ + H]^+^
					131.086	C_10_H_11_	[C_10_H_10_ + H]^+^
					109.065	C_7_H_10_O	[C_7_H_9_O + H]^+^
					105.070	C_8_H_9_	[C_8_H_8_ + H]^+^
					79.054	C_6_H_7_	[C_6_H_6_ + H]^+^
LA-C_1_/C_2_	5.7/5.9	C_31_H_36_O_4_	472.2614	473.2686/473.2687	455.258	C_31_H_35_O_3_	[M-H_2_O + H]^+^
					395.219	C_25_H_31_O_4_	[M-C_6_H_6_ + H]^+^
					338.188	C_22_H_26_O_3_	[M-C_9_H_9_-H_2_O + H]^+^
					245.132	C_19_H_17_	[M-C_12_H_12_-H_8_O_4_ + H]^+^
					219.117	C_17_H_15_	[M-C_14_H_14_-H_8_O_4_ + H]^+^
					193.101	C_15_H_13_	[M-C_16_H_16_ -H_8_O_4_ + H]^+^
					131.086	C_10_H_11_	[C_10_H_10_ + H]^+^
					109.065	C_7_H_10_O	[C_7_H_9_O + H]^+^
					105.070	C_8_H_9_	[C_8_H_8_ + H]^+^
					79.054	C_6_H_7_	[C_6_H_6_ + H]^+^
LA-D_1_	6.2	C_33_H_38_O_4_	498.2770	499.2844	481.2742	C_33_H_37_O_3_	[M-H_2_O + H]^+^
					421.2373	C_27_H_33_O_4_	[M-C_6_H_6_ + H]^+^
					364.203	C_24_H_28_O_3_	[M-C_9_H_9_-H_2_O + H]^+^
					297.163	C_23_H_21_	[M-C_10_H_10_-H_8_O_4_ + H]^+^
					219.117	C_17_H_15_	[M-C_16_H_16_-H_8_O_4_ + H]^+^
					193.101	C_15_H_13_	[M-C_18_H_18_-H_8_O_4_ + H]^+^
					131.086	C_10_H_11_	[C_10_H_10_ + H]^+^
					109.065	C_7_H_10_O	[C_7_H_9_O + H]^+^
					105.070	C_8_H_9_	[C_8_H_8_ + H]^+^
					79.054	C_6_H_7_	[C_6_H_6_ + H]^+^
LA-D_2_	6.4	C_33_H_38_O_4_	498.2770	499.2832	not determined.		

The notorious very poor solubility of laetiporic acids (<1 mg ml^−1^) in organic solvents [5] such as methanol, chloroform, dichloromethane, cyclohexane, butanol, or acetone, combined with the structural similarity of the various compounds prevented recording of unambiguous NMR spectra to confirm structures and, in particular, double bond positions and configurations. For further characterization, we therefore resorted to MS^2^ experiments (Table 1, Supplementary Fig. S3) for seven out of eight metabolites. As expected for β-hydroxy acids, MS^2^ spectra revealed a consistent prevalence for dehydration (*m/z* [M−18 + H]^+^) and decarboxylation (*m/z* [M−44 + H]^+^) during fragmentation of all identified laetiporic acids. Moreover, the elimination of aromatic rings such as benzene (*m/z* 79 [M + H]^+^) by electrocyclic butyl ring contraction provided a defined MS fingerprint for the linear polyene structure of laetiporic acids resulting in MS^2^ fragments of *m/z* [M−78 + H]^+^. This is in agreement with electrocyclic aromatic elimination of toluene and xylene observed in ESI-based MS^2^ fragmentation of polyene-like carotenoids [15, 16].

Beyond these eight laetiporic acids, we detected traces of another set of very minor signals that overlapped with the major peaks. The molecular masses of the latter indicated dehydrated analogs (*m/z* [M−18 + H]^+^) of laetiporic acids A–D to give presumably 2-dehydro-3-deoxylaetiporic acids A–D (*m/z* 403, 429, 455, and 481 [M + H]^+^, respectively). These compounds were produced in amounts that were insufficient for further analysis. Still, a very similar phenomenon was recognized previously when 2-dehydro-3-deoxylaetiporic acid A (Fig. 1) was identified as a side product from *L. sulphureus* [6].

The above findings are remarkable in that one single PKS makes polyenes of (i) different chain length (C_26_–C_32_) and (ii) different degree of hydroxylation. Although precedence exists for variable chain lengths of HR-PKS PPS1 (C_20_–C_22_) of the basidiomycete BY1 [4] and T-toxin (C_35_–C_45_) the high virulence determinant of the ascomycetous maize pathogen *Cochliobolus heterostrophus* [17], the wide spectrum of metabolites produced by LpaA in *A. niger* might be a host-specific effect. Laetiporic acids A_1_ and A_2_ are the major metabolites isolated from the fruiting body or the mycelium of *L. sulphureus* [6]. In contrast, heterologous production predominantly resulted in laetiporic acids B and C (Fig. 3). Therefore*, A. nidulans* FGSC A4 was tested as alternative host and transformed with plasmid pMG49 in which the *lpaA* gene is controlled by the ethanol-inducible, but glucose-repressible alcohol-dehydrogenase promoter (P*alcA*, Supplementary Tables S1, S4 and Supplementary Fig. S1) [18]. The resulting transformant tMG01 and the wild type show comparable growth in presence of d-glucose. However, growth was impeded when ethanol was used as sole carbon source indicating similar toxic effects of laetiporic acids as observed for *A. niger*. When supplemented with 10 mM d-glucose, the mutant showed a strongly retarded growth and its mycelium turned orange to intense red (Fig. 3). Mycelial extracts from tMG01 were analyzed by UHPLC-MS and revealed a similar chromatographic profile as observed for the *lpaA*-expressing *A. niger* mutant tPS02. In contrast, no signals at *λ* = 450 nm were detectable in the parental strain. In summary, these results confirmed laetiporic acids A_1_–D_2_ as products of LpaA and point to an antifungal activity of laetiporic acids when produced in situ.

### Antifungal activity of laetiporic acids

**Fig. 4:**
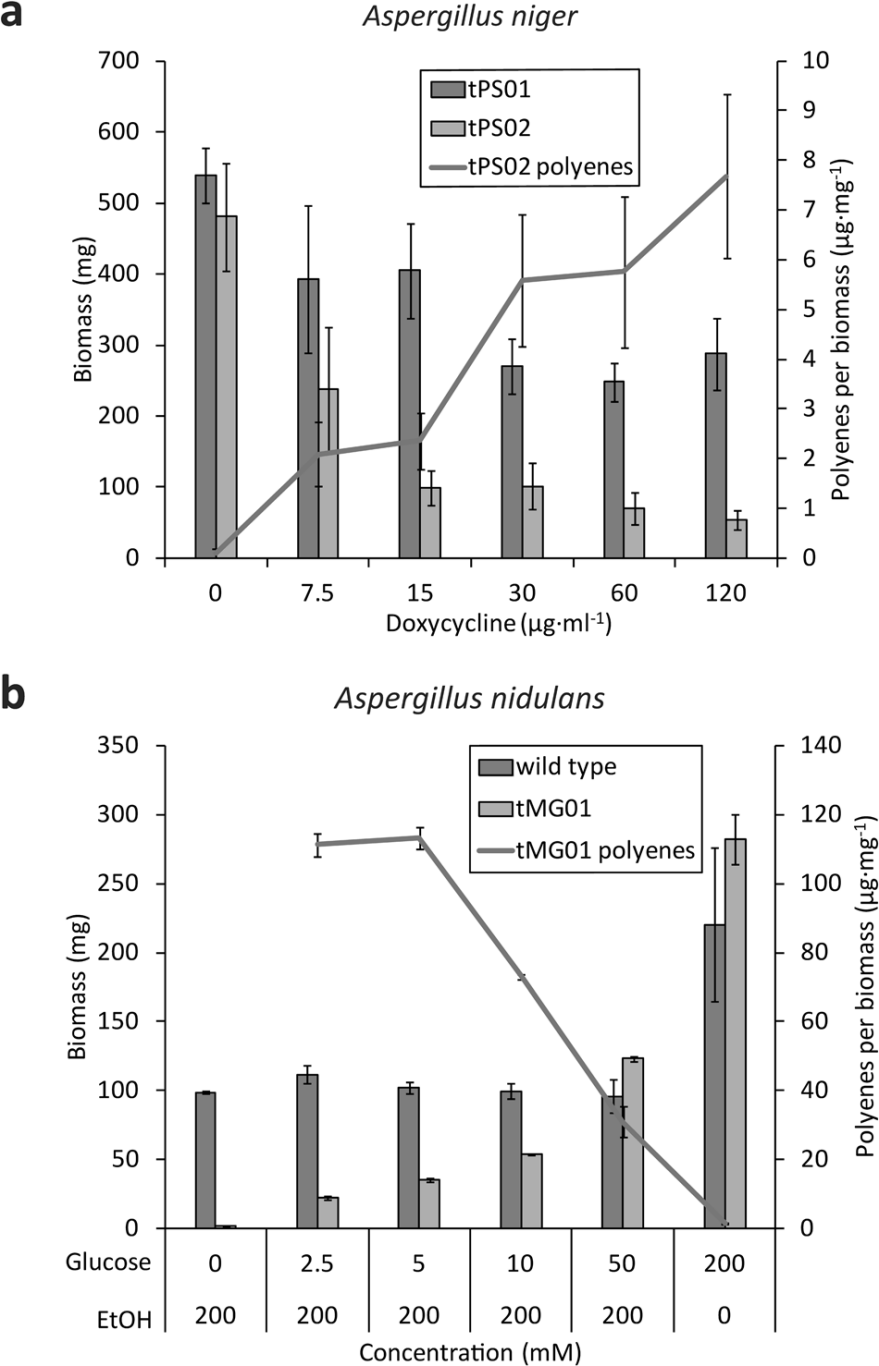
Antifungal activity of laetiporic acids produced in situ by *Aspergillus niger* and *Aspergillus nidulans*. Fungal dry biomass (in mg) was determined for *A. niger* tPS02 (*lpaA*-expressing) and tPS01 (vector control) (**a**) or *A. nidulans* tMG01 (*lpaA*-expressing) and FGSC A4 wildtype strain (**b**), respectively. Polyene production was induced by adding doxycycline (7.5–120 µg ml^−1^) in *A. niger* and repressed by d-glucose (2.5–50 mM) in *A. nidulans*. Cultures without inducer, i.e., doxycycline for *A. niger* or ethanol in *A. nidulans*, served as negative controls. Intracellular polyene concentration is given in µg per mg dry fungal biomass. Polyene production is increased 15-fold in *A. nidulans*, compared to *A. niger*, resulting in a more intense coloration and an antifungal effect (no growth under non-repressing conditions). Hence, polyene content could not be determined under this condition. Bars indicate the standard deviation (*n* = 3)

**Fig. 5:**
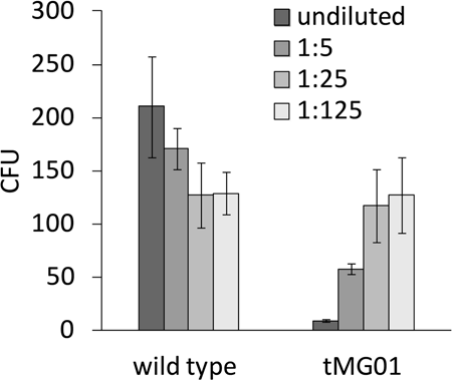
Antifungal activity on *Aspergillus nidulans* protoplasts. Extracts of *A. nidulans* wild type (negative control) and tMG01 were pre-purified, and added undiluted or diluted 1:5, 1:25, and 1:125, respectively, to a protoplast suspension. The undiluted tMG01 extract contained polyenes at a concentration of 4 mg ml^−1^. Bars indicate the standard deviation (*n* = 4); CFU: colony forming units

To follow up on the impeded growth of polyene-producing Aspergilli, we assessed the antifungal activity of the produced cocktail. The agar diffusion assay—a widely used technique in antimicrobial susceptibility testing—relies on water soluble and diffusible substances, which cannot be assumed for laetiporic acids. As an alternative dual strategy, we tested the bioactivity of polyenes (i) in situ during production in *A. niger* and *A. nidulans* (Fig. 4), and (ii) directly on *A. nidulans* protoplasts (Fig. 5). *A. niger* strains tPS01 (control) and tPS02 (*lpaA*-expressing) were cultivated in the absence or presence of 7.5–120 µg ml^−1^ doxycycline to induce gene expression dose-dependently [19]. While doxycycline hardly inhibited growth at 30–120 µg ml^−1^ in the tPS01 control, tPS02 was strongly impaired, and had produced less biomass (Fig. 4). Moreover, growth inhibition correlated with the dose-dependent intrinsic polyene concentration which reached its maximum at 7.5 µg per mg biomass at 120 µg ml^−1^ doxycycline. In a complementary experiment, the *lpaA*-expressing strain *A. nidulans* tMG01 and its parental strain were cultivated in AMM with 200 mM ethanol as carbon source, and variable amounts of glucose (2.5–50 mM) were added prior to inoculation to dose-dependently repress gene expression. Growth of the parental strain was not affected. In contrast, but similarly to what was observed for *A. niger*, growth inhibition was strongest when the highest intrinsic polyene concentration was reached (110 µg mg^−1^ polyenes at 2.5 mM d-glucose). Moreover, without repressing d-glucose (that is, 200 mM ethanol as sole carbon source), spores from tMG01 were arrested in the state of germination, indicating a strong antifungal activity. In a parallel experiment, the LpaA-produced cocktail of multi-chain length laetiporic acids was added to *A. nidulans* wild type protoplasts. Based on the number of colony-forming units, we observed a significantly reduced protoplast viability with undiluted and 1:5 diluted extract, compared to *A. nidulans* wild type extracts (Fig. 5).

## Discussion

Conjugated double bonds are a widespread feature of biologically active natural products, among them the leukotrienes, carotenoids, and clinically used antimicrobially active polyenes, such as nystatin and amphotericin. In the context of basidiomycetes, polyenes are remarkable in that they occur erratically and in taxonomically unrelated groups, among them the Russulales [4] and the Polyporales [5, 6]. Knowledge on the biosynthesis of basidiomycete highly reduced polyketide is scanty. To date, PPS1, the characterized polyene synthase of BY1, a stereaceous basidiomycete [4], is the only reported example. To exclude host-specific effects, we relied on two independent heterologous systems: in one system gene expression is mediated by increasing concentrations of an inductor (doxycycline), while in the second system, the ethanol-mediated induction was attenuable by increasing d-glucose concentrations as repressor. Yet, the polyene profiles were highly congruent, independent of the used strategy.

The combined results from PPS1 and the *Laetiporus* enzyme LpaA reveal common features between these two synthases from unrelated species. Remarkably, polyenes of variable chain length seem to be an intrinsic property of either synthase, yet more pronounced with LpaA, as chain lengths varied between C_26_ and C_32_, while PPS1 produces polyenes with C_20_–C_22_ main chains. Further, double bond shift (i.e., positioned within formal acetate units, rather than between them) is a shared intrinsic property of LpaA and PPS1. In the case of 2-dehydro-3-deoxylaetiporic acid A (Fig. 1), one double bond remains at the canonical α,β-position, although this may be a secondary effect as well and the consequence of water elimination. Finally, either enzyme showed a preference for a particular chain length, in the case of LpaA primarily C_28_ and C_30_ main chains (i.e., laetiporic acids B and C).

Remarkably, LpaA, i.e., a single enzyme, produces an entire set of compounds, which once again supports both the notion of a diversity-oriented secondary metabolism [20] and an emerging concept of multi-product PKSs. Described examples include, e.g., PKS1 of *Colletotrichum lagenarium* that produces tetra- to hexaketides [21] and the *A. terreus* polyketide synthase TerA, which makes tri- to pentaketides [22]. Building upon prior results by Weber et al., we here show that a single enzyme produces four chains, which provides the first layer of diversification and underscores that LpaA has been evolved for diversity, rather than specificity. The second layer to generate diversity is elimination of a formal water molecule from positions C-3 and C-4. Elimination of water is a natural, previously described process [5, 6], which is confirmed by our results. A third dimension to generate diversity is the configuration of double bonds.

Knowledge about the ecological role of basidiomycete polyenes is incomplete, yet inhibition of insect larvae was shown by previous works [1, 2, 4]. Our results on both *Aspergillus* protoplasts and the transgenic Aspergilli add antifungal activity to the biological effects of basidiomycete polyenes. Given the lipophilic character and the length of *Laetiporus* polyenes, activity may be exerted through interference with membranes. However, the assay system included wall-less cells and a production in situ. Therefore, future research is warranted to determine, if intact cell walls protect mycelium from being inhibited. Still, our results confirm the Basidiomycota as prolific producers of antibiotic compounds [23]. Hallmark examples are the agriculturally used fungicides derived from strobilurine [24], or the clinically used derivatives of the antibacterial pleuromutilin [25]. The results presented here contribute to efforts, reflected by this special issue of the Journal of Antibiotics, to understand this aspect of basidiomycete biology more profoundly.

## Supplementary information

Supplemental Material
