# Supplementary material for: The Laetiporus polyketide synthase LpaA produces a series of antifungal polyenes
Source: J Antibiot (Tokyo). 2020 Aug 21;73(10):711–20. doi: 10.1038/s41429-020-00362-6 (PMC7473843; doi:10.1038/s41429-020-00362-6)
Supplement: Supplementary file 1 — Supplemental Material [file 41429_2020_362_MOESM1_ESM.docx]

**Supplementary information**

**The *Laetiporus* polyketide synthase LpaA produces a series of antifungal polyenes**

Paula Sophie Seibold^1^, Claudius Lenz^1^, Markus Gressler^1^, Dirk Hoffmeister^1^

^1^Pharmaceutical Microbiology, Friedrich Schiller University, Jena, Germany

# Table of contents

**Table S1.** Fungal strains. 2

**Table S2.** Oligonucleotides used in this study. 2

**Table S3.** PCR methods**.** 3

**Table S4.** Plasmids used in this study**.** 4

**Table S5.** HPLC methods**.** 4

**Figure S1.** PCR verification of successful *Aspergillus* transformations 5

**Figure S2.** UV/Vis spectra of laetiporic acids A_1_-D_2_ 6

**Figure S3.** Proposed MS² fragmentation patterns of laetiporic acids A_1_-D_2_ 7

**Figure S4.** Comparative MS² fragmentation of laetiporic acid A_1_ and standard 8

**Figure S5.** Chromatographic analysis of light-exposed laetiporic acids 9

**References.**  10

**Table S1.** Fungal strains used in this study.

| **Strain** | **Genotype** | **Reference** |
| --- | --- | --- |
| *L. sulphureus* JMRC SF012599 | wild type | This study |
| *Aspergillus niger* ATNT16Δ*pyrG*x24 | TetOn:*terR*_*ble*; Δ*pyrG*::*ptrA* | [1] |
| *Aspergillus niger* tPS01 | TetOn:*terR*_*ble*; Δ*pyrG*::*ptrA; PterA:His6_pyrG* | This study |
| *Aspergillus niger* tPS02 | TetOn:*terR*_*ble*; Δ*pyrG*::*ptrA; PterA:His6:lpaA_pyrG* | This study |
| *Aspergillus nidulans* FGSC A4 | wild type | FGSC |
| *Aspergillus nidulans* tMG01 | P*alcA:lpaA_ptrA* | This study |

**Table S2.** Oligonucleotides used in this study.

| Name | Sequence (5´-3´) | Target | Purpose |
| --- | --- | --- | --- |
| oCL46 | ATGGCAGTGAGGCCGCCCAGCTAC | *L. sulphureus* *lpaA* | cloning of *lpaA* CDS in pJET1.2 (pCL10) |
| oCL47 | TCATGCCACAATGCCGTTCAATATCTC | *L. sulphureus* *lpaA* | cloning of *lpaA* CDS in pJET1.2 (pCL10) |
| oMG457 | GTGATGATGATGACTAGTCATGGTGCTGTGATGAGAAGTTTG | pSMX2-URA | introduces *PacI* sites in pSMX2-ura (pPS01) |
| oMG458 | CATCACCATTTAATTAACTAGCAGCAGTGATTTCAATCTGAACC | pSMX2- URA | introduces *PacI* sites in pSMX2-ura (pPS01) |
| oMG459 | CATCACCATTTAATTAACATGGCAGTGAGGCCGCCCAG | pCL10 (*lpaA*) | cloning of *lpaA* CDS in pPS01 (pPS03) |
| oMG460 | ATCACTGCTGCTAGTTAATTAATCATGCCACAATGCCGTTC | pCL10 (*lpaA*) | cloning of *lpaA* CDS in pPS01 (pPS03) |
| oMG370 | GATCCTCTCTCTGATATTGTCG | pPS01/pPS03 | integration of *lpaA* in *A. niger* genome |
| oMG116 | GAGATGTGGTAGACGATTGATCC | pPS01/pPS03 | integration of *lpaA* in *A. niger* genome |
| oMG468 | ACTTAACGTTACTGAAATCATCAAACAG | pMD03 | amplifies expression vector pMD03 |
| oMG469 | TTTGAGGCGAGGTGATAGGATTG | pMD03 | amplifies expression vector pMD03 |
| oMG471 | TGATTTCAGTAACGTTAAGTTCATGCCACAATGCCGTTCAATATC | pCL10 (*lpaA*) | cloning of *lpaA* CDS in pMD03 (pMG49) |
| oMG472 | TCCTATCACCTCGCCTCAAAATGGCAGTGAGGCCGCCCAG | pCL10 (*lpaA*) | cloning of *lpaA* CDS in pMD03 (pMG49) |
| oCL35 | GCATAGCGTTTGGCTTGATAC | pMG49 | integration of *lpaA* in *A. nidulans* genome |
| oMG474 | CATCCCCGCATAGCTGAACATC | pMG49 | integration of *lpaA* in *A. nidulans* genome |

**Table S3.** PCR methods.

| **Method** | **20 µl Reaction Mix** | | **Thermal cycling*** | |
| --- | --- | --- | --- | --- |
|  | **Component** | **Volume (µl)** | **Temperature (°C)** | **Time** |
| A | 5x Phusion HF enzyme buffer  Primer forward 10 pmol·µl^-1^  Primer reverse 10 pmol·µl^-1^  10 mM dNTP mix  DNA template (100 ng·µl^-1^)  Phusion DNA Polymerase  dH_2_O | 4.0  1.0  1.0  0.4  1.0  0.1  12.5 | 98  98  60  72  72  12 | 2 min  30 sec  30 sec  4.5 min  5 min  ∞ |
| B | 5x Phusion HF enzyme buffer  Primer forward 10 pmol·µl^-1^  Primer reverse 10 pmol·µl^-1^  10 mM dNTP mix  DNA template (50 ng·µl^-1^)  DMSO  Phusion DNA Polymerase  dH_2_O | 4.0  1.0  1.0  0.4  0.1  1.5  0.1  11.9 | 98  98  60  72  72  12 | 2 min  20 sec  20 sec  5 min  10 min  ∞ |
| C | 5x Phusion HF enzyme buffer  Primer forward 10 pmol·µl^-1^  Primer reverse 10 pmol·µl^-1^  10 mM dNTP mix  DNA template (500 ng·µl^-1^)  DMSO  Phusion DNA Polymerase  dH_2_O | 4.0  1.0  1.0  0.4  0.5  1.5  0.1  11.5 | 98  98  60  72  72  12 | 2 min  20 sec  20 sec  5 min  10 min  ∞ |
| D | 10x DreamTaq enzyme buffer  Primer forward 10 pmol·µl^-1^  Primer reverse 10 pmol·µl^-1^  10 mM dNTP mix  DNA template (500 ng·µl^-1^)  DMSO  DreamTaq Polymerase  dH_2_O | 2.0  0.4  0.4  0.4  0.5  1.5  0.1  14.7 | 95  95  60  72  72  12 | 2 min  20 sec  20 sec  2 min  5 min  ∞ |

* Methods A-C: 35 cycles, method D: 31 cycles.

Method A: **amplification of *lpaA***

oligonucleotides: cCL46, oCL47

template: first strand reaction of *lpaA* reverse transcription.

Method B: **incorporation of *Pac*I restriction site into pSMX2‑URA**

oligonucleotides: oMG457, oMG458

template: pSMX2-URA

**addition of *Pac*I restriction sites to flank the *lpaA* gene**

oligonucleotides: oMG459, oMG460

template: pCL10

**amplification of a vector backbone** **for alcohol-inducible *lpaA* expression**

oligonucleotides: oMG468, oMG469

template: pMD03

**amplification of *lpaA* for an alcohol-inducible *lpaA* expression construct**

oligonucleotides: oMG471, oMG472

template: pCL10

Method C: **verification of full-length integration of *lpaA* into the *A. niger* genome**

oligonucleotides: oMG370, oMG116

template: *A. niger* gDNA

Method D: **verification of integration of *lpaA* into the *A. nidulans* genome**

oligonucleotides: oCL35, oMG474

template: *A. nidulans* gDNA

**Table S4.** Plasmids used in this study.

| **Plasmid name** | **Vector backbone** | **Gene product** | **His_6_-tag** | **Reference** |
| --- | --- | --- | --- | --- |
| pCL10 | pJET1.2 | LpaA | no | This study |
| pSMX2-URA | pUC19 | -* | yes | [1] |
| pPS01 | pSMX2-URA | -* | yes | This study |
| pPS03 | pPS01 | His_6_:LpaA | yes | This study |
| pMD03 | pUC19 | mCherry | no | [2] |
| pMG49 | pMD03 | LpaA | no | This study |

* only codons for hexahistidine tag

**Table S5.** HPLC methods (FA: formic acid).

| **Method** | **Column** | **Parameters** | **Eluents** | **Gradient** |
| --- | --- | --- | --- | --- |
| A | ZORBAX EclipsePlus C18 2.1 x 50 mm, 1.8 µm | T: 30 °C  flow: 1 ml min^-1^ | A: water + 0.1% FA  B: acetonitrile | 0 min: 5% B  3 min: 50% B  7 min: 75% B  8 min: 100% B |
| B | ZORBAX EclipsePlus C18 2.1 x 50mm, 1.8 µm | T: 30 °C  flow: 1 ml min^-1^ | A: water + 0.1% FA  B: acetonitrile | 0 min: 5% B  0.5 min: 5% B  1 min: 75% B  2 min: 100% B |
| C | Agilent Eclipse XDB C18  9.4 x 250 mm, 5 µm | T: 12 °C  flow: 2.5 ml min^-1^ | A: water + 0.1% FA  B: acetonitrile | 0 min: 70% B  5 min: 80% B  16 min: 88 % B  18 min: 100% B |
| D | Agilent Eclipse XDB C18  9.4 x 250 mm, 5 µm | T: 12 °C  flow: 2.5 ml min^-1^ | A: water + 0.1% FA  B: acetonitrile | 0 min: 70% B  5 min: 84% B  16 min: 92 % B  18 min: 100% B |
| E | Agilent Eclipse XDB C18  9.4 x 250 mm, 5 µm | T: 12 °C  flow: 2.5 ml min^-1^ | A: water*  B: acetonitrile | 0 min: 25% B  2 min: 25% B  25 min: 50 % B  27 min: 100% B |
| F | Agilent Eclipse XDB C18  9.4 x 250 mm, 5 µm | T: 12 °C  flow: 2.5 ml min^-1^ | A: water*  B: acetonitrile | 0 min: 30% B  2 min: 30% B  25 min: 55 % B  27 min: 100% B |

* adjusted to pH 8 with NH_3_.

**Figure S1**. Agarose gels of polymerase chain reactions that verify successful *Aspergillus* transformations. Panel a: *A*. *niger* tPS01 (vector control, expected PCR product size 1067 bp), the untransformed parental strain (negative control), and seven transformants of tPS02 (*lpaA*-integration, expected PCR product size 9272 bp). Plasmid pPS03 served as positive control. Panel b: *A*. *nidulans* FGSC A4 (wild type, untransformed parental strain as negative control), and seven transformants of tMG01 (*lpaA*-integration, expected PCR product size 1980 bp). Plasmid pMG49 is the positive control. Asterisks indicate the transformants chosen for further work.

**Figure S2**. UV/VIS spectra of laetiporic acids A_1_-D_2_ isolated from *lpaA*-expressing *A. niger* tPS02. The local maxima of the wavelength of highest absorbance are indicated in the respective panels.


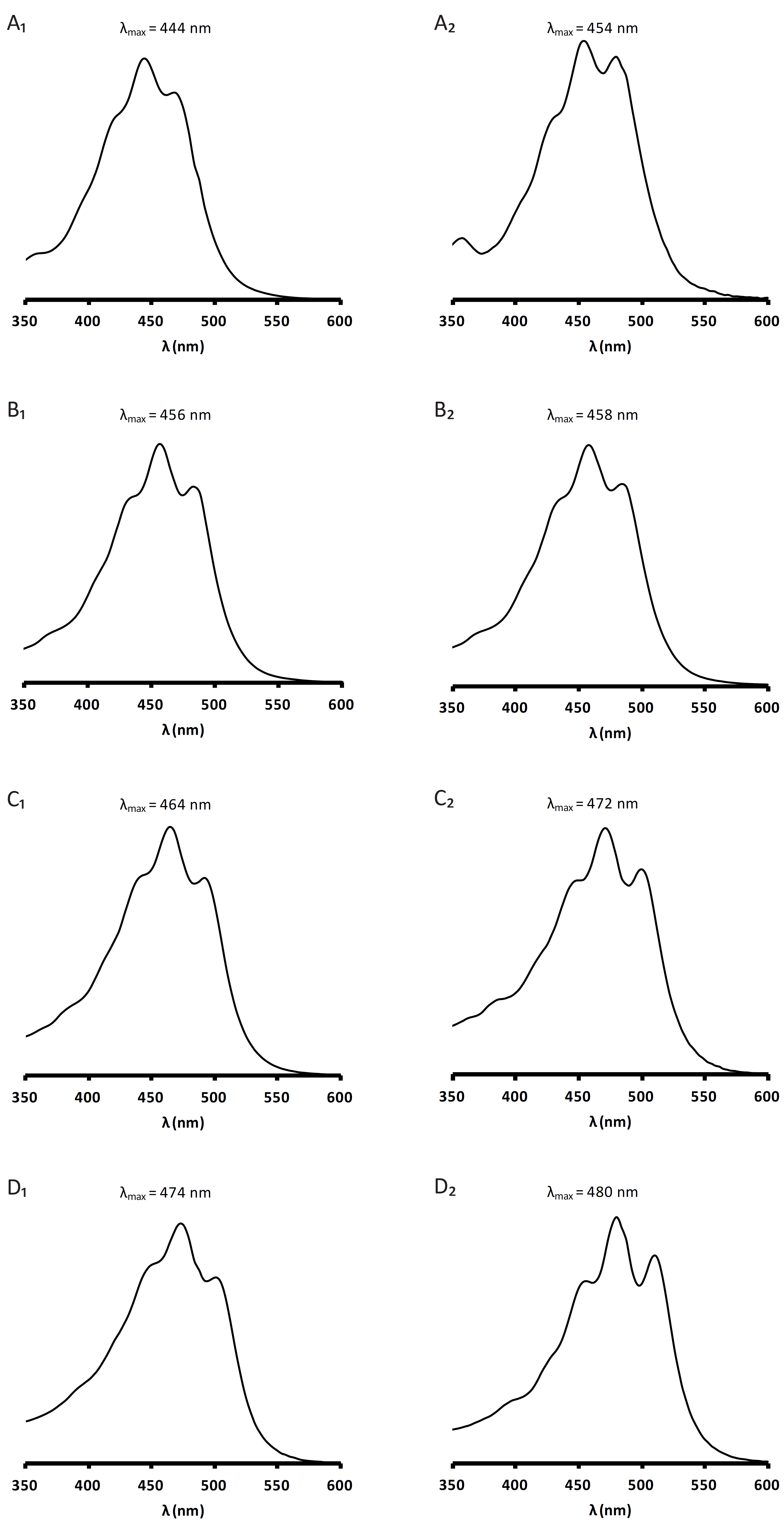


**Figure S3**. Proposed MS^2^ fragmentation pattern of laetiporic acid A. Ion masses that were identified in the MS² spectra are highlighted in blue.
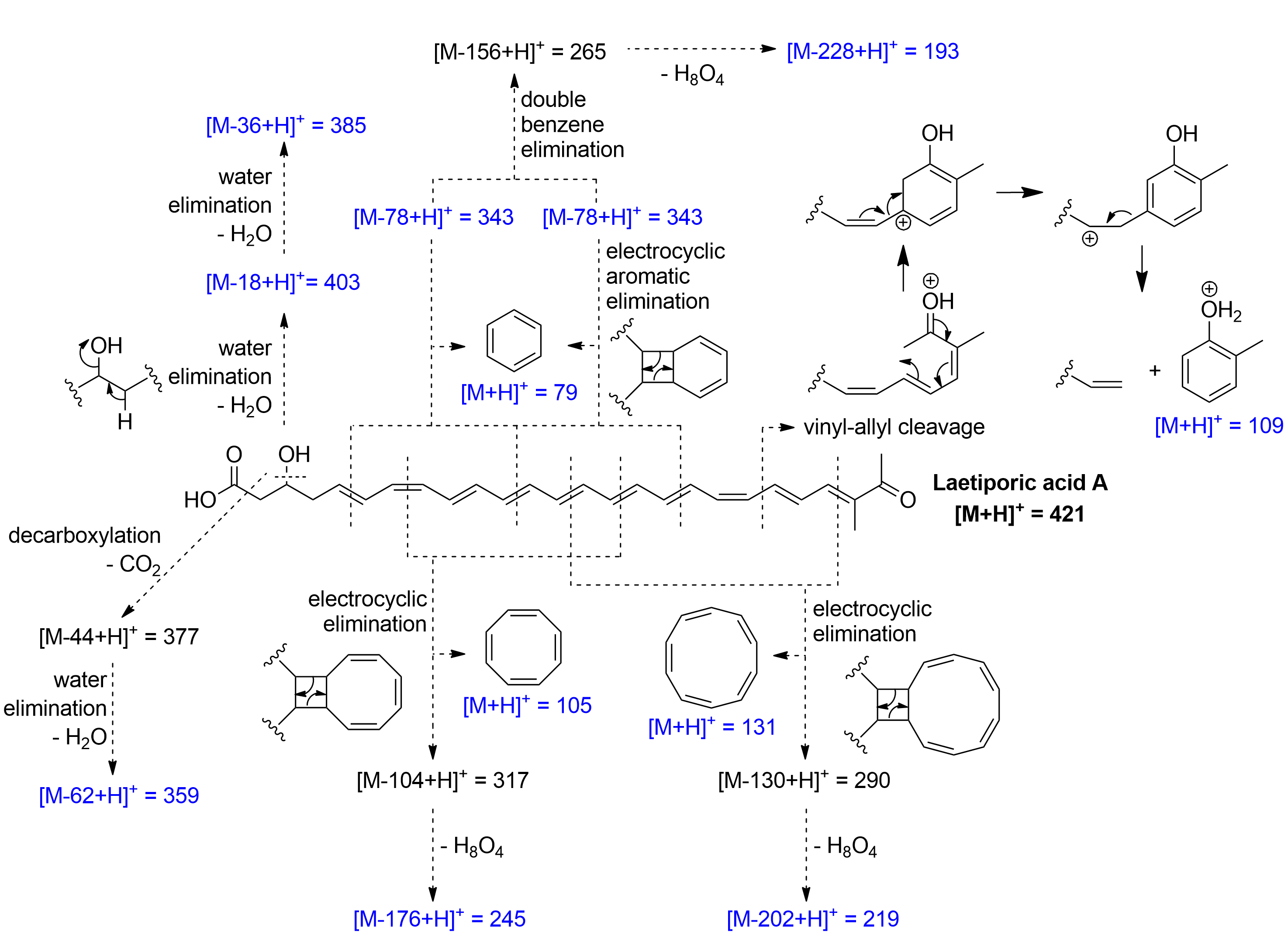


**Figure S4**. Comparative MS^2^ fragmentation of a laetiporic acid A_1_ standard, isolated from *Laetiporus sulphureus* (panel a), and laetiporic acid A_1_ produced by *Aspergillus nidulans* tMG01 (panel b). Bold signal labels indicate fragments suggested in above Figure S3.





**Figure S5**. Chromatographic comparison of laetiporic acids exposed to light for 24 h or kept in the dark. Chromatograms were extracted at λ=450 nm.

**References**

1. Geib E, Baldeweg F, Doerfer M, Nett M, Brock M. Cross-Chemistry Leads to Product Diversity from Atromentin Synthetases in Aspergilli from Section Nigri. *Cell Chem Biol*. 2019;26:223-34.
2. Dörfer M, et al. Melleolides impact fungal translation via elongation factor 2. *Org Biomol Chem*. 2019;17:4906-16.
